# Dr. Charles J. Bacon

**Published:** 1914-12

**Authors:** 


**Dr. Charles J. Bacon,** Albany 1865, died at his home in Syracuse September 3, aged 70. He was formerly in practice in Fulton, where he founded the Lee Memorial Hospital and was for many years its president. He was also Consulting Physician to the Oswego Hospital. He came to Syracuse in 1910.

